# Ferroptosis in bovine mastitis: multidimensional mechanisms, stratified assessment, and intervention prospects

**DOI:** 10.3389/fvets.2026.1874206

**Published:** 2026-06-30

**Authors:** Lingling Cheng, Gaojie Song

**Affiliations:** 1 College of Veterinary Medicine, Gansu Agricultural University, Lanzhou, Gansu, China; 2 Jiangxi Provincial Key Laboratory of Cell Precision Therapy, School of Basic Medical Sciences, Jiujiang University, Jiujiang, Jiangxi, China

**Keywords:** blood–milk barrier, bovine mastitis, ferroptosis, lipid peroxidation, stratified assessment

## Abstract

Mastitis remains a major constraint on dairy cow health and production efficiency. Even after pathogen clearance, inflammation, epithelial injury, and lactation impairment can persist, indicating that disease outcome is not determined solely by pathogen burden. Ferroptosis, a regulated form of cell death driven by iron-dependent membrane phospholipid peroxidation, provides a mechanistic framework that links iron dyshomeostasis, oxidative injury, and irreversible tissue damage. Current evidence suggests that increased oxidative load, altered iron flux, restriction of the cysteine–glutathione (GSH)–glutathione peroxidase 4 (GPX4) axis, and remodeling of the membrane-lipid substrate pool can jointly lower the ferroptotic threshold of mammary epithelial cells in the mastitic microenvironment. Within defined pathogen contexts and temporal windows, oxidized lipids and damage-associated molecular patterns (DAMPs) may further contribute to inflammatory amplification, blood–milk barrier disruption, and lactation decline. However, direct *in vivo* causal evidence in bovine mastitis remains limited. Ferroptosis further intersects with pyroptosis, necroptosis, and other regulated cell-death pathways, underscoring its strong context dependence. This review synthesizes current evidence on the core mechanisms of ferroptosis, its triggers in the mastitic microenvironment, the translation of molecular injury into tissue dysfunction, evidence standards for causal attribution, boundaries of contribution, and prospects for stratified assessment and intervention. In sum, ferroptosis is best regarded as a candidate framework for host-damage amplification in mastitis rather than a universally established execution pathway. Advancing its translational value will require spatiotemporally resolved evidence, cell-type specificity, and causal rescue experiments.

## 1 Introduction

Bovine mastitis has a high incidence, causes substantial economic losses, and has long-term implications for animal welfare, making it a central challenge in dairy herd health management (1). Its impact is not limited to infection itself, but also includes persistent mammary epithelial injury and impaired lactation (2). Antibiotic-centered therapy can reduce bacterial burden and improve bacteriological cure rates; however, in many non-severe cases, inflammatory resolution and functional recovery do not proceed in parallel, and reductions in somatic cell count (SCC) or recovery of milk yield are not always achieved simultaneously (3). Clinical observations likewise show that inflammation and mammary dysfunction may persist even after pathogen clearance (4). These findings suggest that disease outcome may also depend on host-damage amplification processes that are independent of, or lag behind, pathogen clearance.

Conventional models of mastitis pathogenesis emphasize innate immune recognition triggered by pathogen-associated molecular patterns, followed by inflammatory cascades and intensified oxidative stress (5). This framework alone, however, does not fully explain the heterogeneous outcomes observed in some clinical and subclinical settings. For example, in some subclinical or protracted cases, milk oxidative-stress markers and metabolic abnormalities are markedly elevated, whereas recovery of milk yield and lactose content is incomplete or asynchronous (2, 6). If these changes are interpreted simply as increased reactive oxygen species (ROS), the links among oxidative stress, membrane injury, inflammatory signaling, and cell death remain difficult to test mechanistically. A framework is therefore needed that integrates membrane lipid peroxidation, iron dyshomeostasis, and failure of specific antioxidant defenses. Ferroptosis provides such a framework. It is driven by iron-dependent phospholipid peroxidation and is regulated by the cystine/glutamate antiporter system x_c^−^ (System x_c^−^)–glutathione (GSH)–glutathione peroxidase 4 (GPX4) axis and lipid-remodeling pathways such as acyl-CoA synthetase long-chain family member 4 (ACSL4) (7). Studies in bovine mammary epithelial systems have reported ferroptosis-consistent changes, including enhanced lipid peroxidation, remodeled iron homeostasis, and impaired GPX4/GSH defenses. Evidence from selected pathogen-related mastitis models further shows that pathogen stimulation can be accompanied by iron accumulation, enhanced lipid peroxidation, and disruption of GPX4-related defenses, supporting a linked process in which declining antioxidant reserve, altered iron availability, and rising membrane-lipid peroxidation pressure converge (8, 9).

This review systematically evaluates the hypothesis that ferroptosis contributes to damage amplification in bovine mastitis. The central question is: under which pathogen backgrounds, temporal windows, and cell types might ferroptosis-related events act as candidate mechanisms of host-damage amplification, rather than being presumed to represent a universal dominant pathway? To address this question, the review first discusses the core mechanistic basis of ferroptosis, then examines how the mastitic microenvironment lowers its activation threshold, how ferroptosis-related injury may be translated into inflammatory amplification and functional impairment, and how more robust causal evidence can be established in complex disease models. To maintain a consistent evidentiary standard throughout the review, the following box outlines the evidence framework adopted here.

The evidence discussed in this review derives from different experimental levels, including *in vitro* bovine mammary epithelial cell systems, *ex vivo* or clinical mastitis tissue observations, *in vivo* infection models, and naturally occurring bovine mastitis cases. These evidence levels are not equivalent in causal strength. Direct *in vivo* causal evidence in bovine mastitis remains limited, and many mechanistic links still rely on bovine mammary epithelial cell models or selected pathogen-related experimental systems. Therefore, this review distinguishes model-based evidence from natural-case evidence and avoids treating ferroptosis as a universal cause across all mastitis cases.

Box 1 **Evidence framework for establishing the contribution of ferroptosis in mastitis.**

To avoid equating generic oxidative injury, lipid peroxidation phenotypes, or terminal cell disintegration with ferroptosis, this review evaluates the mechanistic contribution of ferroptosis in mastitis using a three-tier framework: convergent evidence, causal validation, and contextual control. The goal is not to raise the evidentiary threshold artificially, but to ensure that claims are aligned with the strength of the supporting evidence.

- Convergent evidence. Ferroptosis should not be inferred from a single marker. Instead, three categories of readouts should show concordant support: first, iron-dependent alterations, such as an increased labile iron pool (LIP), enhanced ferritinophagy, or mitigation of injury by iron chelation; second, phospholipid-level peroxidation, rather than only increased total reactive oxygen species (ROS) or malondialdehyde (MDA); and third, impairment of key defense axes, such as restriction of glutathione (GSH)/glutathione peroxidase 4 (GPX4)/solute carrier family 7 member 11 (SLC7A11), spatially and temporally aligned with lipid peroxidation.
- Causal validation. On the basis of convergent evidence, functional rescue is needed to increase claim strength. A more reliable approach is to combine interventions with distinct mechanisms, such as lipid-radical scavengers and iron chelators, and to determine whether they produce consistent protection. Genetic manipulation of key nodes, such as GPX4, ACSL4, or nuclear receptor coactivator 4 (NCOA4), can further strengthen causal inference.
- Contextual control. In a complex disease model such as mastitis, pathogen type, sampling time, drug exposure, tissue compartment, and parallel regulated cell-death pathways should be reported in parallel to avoid misclassifying mixed injury as ferroptosis-dominant damage.

Accordingly, when only convergent evidence is available, wording should be limited to “suggests,” “is consistent with ferroptosis,” or “ferroptosis may participate.” When convergent evidence is accompanied by consistent causal rescue, it is more appropriate to state that ferroptosis makes a necessary contribution to tissue injury. Only after contextual control has been achieved should ferroptosis be discussed, within defined pathogen, temporal, and cellular contexts, as one of the principal execution pathways or as a feasible intervention target.

## 2 Core mechanisms of ferroptosis: potential intersections with mammary physiology

This section summarizes the core mechanistic modules of ferroptosis and their intersections with the physiological background of the lactating mammary gland. Ferroptosis is a regulated cell-death modality centered on iron-dependent accumulation of membrane phospholipid peroxides and constrained by metabolic and antioxidant thresholds (7). Its occurrence depends on the imbalance of three interlinked homeostatic systems: the iron-metabolic network that supplies catalytic iron, the membrane-lipid environment that determines peroxidation susceptibility, and the GPX4-centered system that clears phospholipid peroxides (10). When lipid peroxide generation persistently exceeds clearance capacity, local fluctuations can shift from reversible perturbations to chain amplification, ultimately resulting in membrane-injury-driven terminal outcomes. Thus, ferroptosis is best understood through the dynamic balance among iron, lipid substrates, and antioxidant defenses, rather than through ROS accumulation alone (11–13).

**Figure 1:**
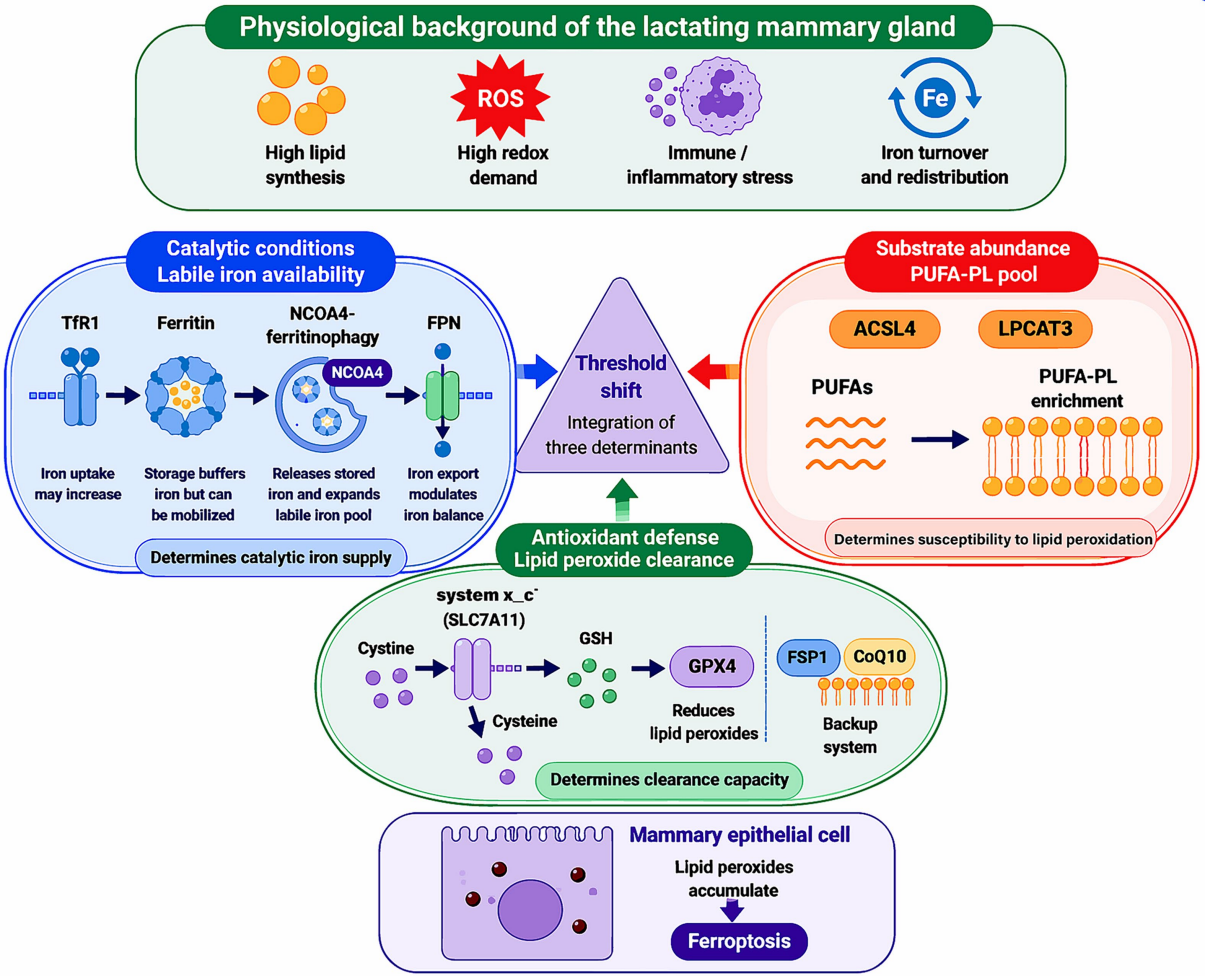
Intersections between core ferroptosis modules and the physiological background of the lactating mammary gland. This figure summarizes the three-part framework through which mammary epithelial cells may enter a ferroptosis-susceptible state. Iron homeostasis determines the availability of catalytically reactive iron; the lipid-substrate module determines the peroxidation susceptibility of membrane phospholipids; and antioxidant defenses determine the capacity to clear phospholipid peroxides. High lipid synthesis, high redox demand, and iron turnover in the lactating mammary gland may together shape a lower ferroptotic threshold, whereas infection- and inflammation-related pressures may further drive this susceptible state toward pathological injury.

The lactating mammary gland provides a distinctive physiological setting in which these modules can converge. Active lipid synthesis and membrane trafficking alter phospholipid composition and increase the abundance of polyunsaturated fatty acid-containing phospholipids (14, 15), whereas high metabolic and inflammatory pressure increases the consumption of reducing equivalents such as GSH (16). At the same time, infection-induced immune-cell infiltration and tissue injury can alter local iron distribution and labile iron-pool dynamics through iron redistribution, disrupted iron storage and release, and Fe^2+^ accumulation in parenchymal cells (8, 9, 17). These changes do not themselves prove that ferroptosis is occurring, but they can lower the ferroptotic threshold of mammary epithelial cells and increase their vulnerability under pathological stress (8, 9). The following subsections examine these three modules as a mechanistic basis for assessing ferroptosis in mastitis (Figure 1).

### 2.1 Iron homeostasis: formation and dynamic regulation of the catalytic core

Ferroptosis is “iron-dependent” primarily because ferrous iron can catalyze chain lipid peroxidation through Fenton chemistry. The size of the intracellular labile iron pool (LIP) is therefore a central determinant of ferroptosis susceptibility (17). The LIP is not static; it is jointly regulated by iron uptake, storage, mobilization, and export. Among these processes, transferrin receptor 1-mediated uptake is a major input route and may be upregulated under inflammatory or other stress conditions, thereby increasing iron influx (7).

Ferritin is the main intracellular iron-storage protein, and its basic function is to sequester iron and limit LIP expansion. Nuclear receptor coactivator 4 (NCOA4)-mediated ferritinophagy directly links iron storage with LIP expansion: after ferritin is targeted to lysosomes for degradation, stored iron is released again (18, 19). In bovine mastitis, this process is not merely theoretical. Recent work shows that *Klebsiella pneumoniae* can promote iron release through NCOA4-mediated ferritinophagy, induce ferroptosis, and aggravate lactation dysfunction, indicating that an iron-buffering reservoir can become a pro-oxidant iron source in this context (20). In addition, the status of iron-export proteins also affects LIP homeostasis (21). Thus, iron homeostasis in mastitis is best interpreted as a dynamic circuit rather than through changes in any single molecule.

### 2.2 Lipid peroxidation: execution of specific injury and substrate determination

The executional event of ferroptosis is peroxidation of membrane phospholipids containing polyunsaturated fatty acids (PUFA-PLs), and its specificity is largely determined by membrane-lipid composition (22). Acyl-CoA synthetase long-chain family member 4 (ACSL4) and lysophosphatidylcholine acyltransferase 3 (LPCAT3) are key enzymes that regulate membrane-lipid composition. Together, they drive the incorporation of long-chain polyunsaturated fatty acids (PUFAs) into membrane phospholipids, especially phosphatidylethanolamine, thereby creating a lipid-substrate pool that is highly susceptible to peroxidation. ACSL4/LPCAT3 expression therefore strongly influences cellular sensitivity to ferroptosis ACSL4 dictates ferroptosis sensitivity by shaping cellular lipid composition (10, 23).

When sufficient catalytic iron is available from the LIP, membrane PUFA-PLs become substrates for non-enzymatic chain lipid peroxidation; in some settings, lipoxygenases may further accelerate this process (24, 25). In the lactating mammary gland, mammary epithelial cells are highly active lipid-synthetic cells, and their membrane systems contain relatively abundant PUFA-PLs. This provides favorable conditions for the initiation and propagation of peroxidation reactions (26). Once antioxidant defenses fail, lipid peroxidation can accumulate rapidly and propagate within susceptible membrane compartments (22).

### 2.3 Antioxidant defenses: threshold control at the point of fate transition

Whether a cell survives after lipid peroxidation begins depends on whether its antioxidant defenses can efficiently clear phospholipid hydroperoxides and interrupt chain reactions. The core of this defense is the cystine/glutamate antiporter system x_c^−^–glutathione (GSH)–glutathione peroxidase 4 (GPX4) axis. System x_c^−^ imports cystine in exchange for glutamate and thereby supports intracellular GSH synthesis; GSH is an essential cofactor for GPX4; and GPX4 directly reduces phospholipid hydroperoxides, acting as the terminal executor of lipid-peroxide detoxification. Weakening of any component of this axis, including cystine deprivation, GSH depletion, or GPX4 inactivation, can directly induce ferroptosis (18, 22, 27).

In the mastitic microenvironment, this defense system faces multiple pressures. General mechanistic studies show that inflammatory stress can restrict cystine supply through the cystine/glutamate antiporter subunit SLC7A11/xCT and weaken GPX4 defenses, thereby reducing cellular anti-peroxidation reserve (28). In mastitis-related epithelial or host–pathogen models, pathogen stimulation has been associated with SLC7A11 suppression, GSH/GPX4-axis impairment, and increased lipid peroxidation; *Staphylococcus aureus*-related models further suggest regulation through sirtuin 1 (SIRT1)/p53/SLC7A11 signaling (29). At the same time, respiratory bursts from immune cells accelerate GSH consumption, whereas iron loading increases the rate of lipid-peroxide generation, jointly pushing the defense system toward failure (8, 9). In addition, the ferroptosis suppressor protein 1 (FSP1)-coenzyme Q10 (CoQ10) axis and other backup systems underscore cellular heterogeneity and the non-exclusivity of GPX4. The fate of mammary epithelial cells should therefore be evaluated by considering both the core axis and auxiliary defense systems (27, 30).

### 2.4 Mechanistic convergence: why the mammary gland may be ferroptosis-susceptible

The relevance of ferroptosis to mastitis lies in the convergence of iron metabolism, lipid-substrate availability, and antioxidant capacity within a tissue that is already metabolically active and structurally dependent on membrane integrity. In the lactating mammary gland, active lipid synthesis and membrane trafficking enrich the epithelial membrane system with phospholipid substrates that can undergo peroxidation (31). At the same time, lactation, metabolic activity, and immune responses place sustained demands on reducing resources such as GSH (16, 32). The gland also requires continuous iron handling, and shifts in local iron homeostasis can influence the labile iron pool (33). These features do not indicate that ferroptosis is constitutively active in the mammary gland; rather, they define a physiological background in which the ferroptotic threshold can be approached more readily when pathological stress is imposed.

This convergence becomes particularly relevant during mastitis. Infection and inflammation can increase oxidative pressure, redistribute iron, and restrict antioxidant defenses within the same temporal window. Under these conditions, membrane phospholipid peroxidation is more likely to exceed clearance capacity and to affect not only cell survival, but also tight-junction integrity, barrier permeability, secretory homeostasis, and local inflammatory responses (34, 35). The mammary gland should therefore be viewed not as an organ in which ferroptosis inevitably occurs, but as a tissue in which lactation-associated physiology creates a susceptible background that mastitic stress may push toward pathological injury.

Susceptibility may also vary across the lactation cycle. Early lactation and the transition period involve abrupt secretory activation, metabolic stress, immune adjustment, and a higher risk of intramammary infection, whereas peak lactation imposes increased secretory and lipid-synthetic demand on mammary epithelial cells (36, 37). The dry period also represents a vulnerable window because mammary involution, epithelial remodeling, teat-end changes, and new intramammary infections can reshape inflammatory and iron-handling states (38). These stage-specific features do not establish stage-specific ferroptosis, but they provide a physiological basis for time-window-specific studies of ferroptosis-related susceptibility in bovine mastitis.

## 3 The mastitic microenvironment as a driver of ferroptosis: oxidative stress, iron flux, and substrate-pool remodeling

**Figure 2:**
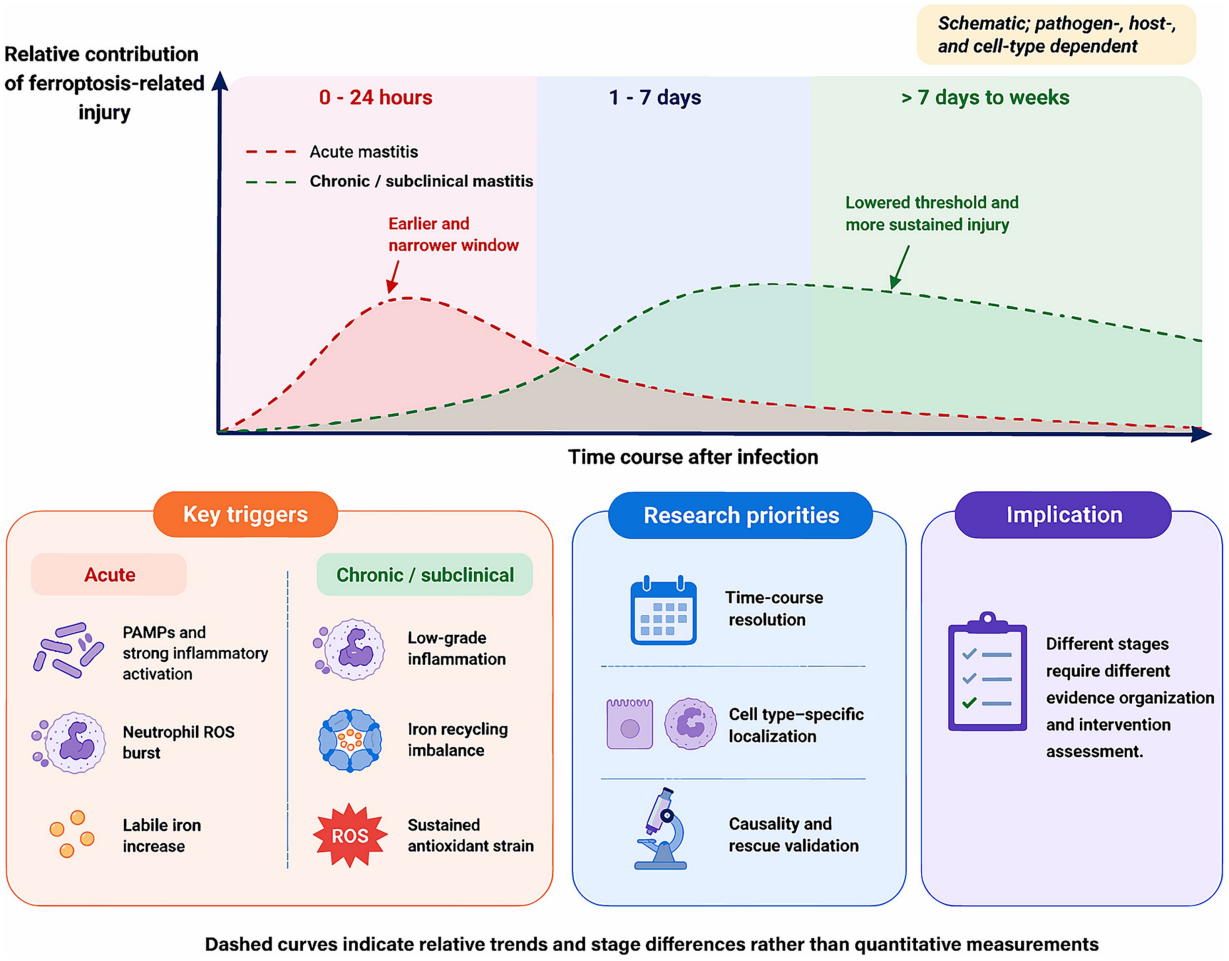
Conceptual temporal windows of ferroptosis-related injury in acute and chronic/subclinical mastitis. This figure provides a conceptual synthesis of possible temporal patterns of ferroptosis-related injury in acute and chronic or subclinical mastitis, based on direct evidence, indirect evidence, and mechanistic inference. The curves indicate relative trends and stage differences only; they do not represent quantitative activity and should not be interpreted as fixed kinetic patterns fully validated in natural bovine cases.

Building on the core mechanistic modules described in Section 2, this section focuses on how the mastitic microenvironment engages these same axes through oxidative stress, iron-flux remodeling, and metabolic perturbation. Ferroptosis-related injury in mastitis is rarely driven by a single stimulus. It is more likely to arise from multiple disturbances that converge within the local pathological microenvironment. During infection and inflammation, mammary tissue is exposed to increased oxidative load, restricted reducing-substrate supply, remodeled iron flux, and altered lipid metabolism. This interpretation is supported by pathogen-related mammary epithelial studies and recent veterinary ferroptosis literature, which together highlight infection-associated oxidative stress, regulated cellular stress responses, iron-dependent lipid peroxidation, and antioxidant-defense failure in animal disease contexts (39, 40). When these pressures coincide, lipid peroxide generation can persistently exceed phospholipid peroxide clearance. Oxidative fluctuations that would otherwise remain buffered may then shift toward threshold instability, resulting in membrane damage and barrier dysfunction. Current evidence suggests that the contribution of ferroptosis in mastitis is strongly context- and stage-dependent: upstream triggers, kinetics, and relationships with other cell-death pathways may differ by pathogen background, disease phase, and cellular compartment. Rather than assuming a single pathway operating throughout the disease course, it is more informative to analyze how inflammatory signaling, iron flux, and metabolic remodeling lower the ferroptotic threshold in the mastitic microenvironment. A conceptual summary of acute versus chronic or subclinical stages is shown in Figure 2.

### 3.1 Pathogens and PAMPs: inflammatory signaling, oxidative burden, and antioxidant erosion

After pathogen-associated molecular patterns (PAMPs) are recognized by epithelial and immune cells, signaling converges on hubs such as nuclear factor-kappa B (NF-κB) and mitogen-activated protein kinase (MAPK), inducing inflammatory mediators and reshaping the cellular stress state (41–43). For ferroptosis, the key issue is not merely whether inflammation is activated, but whether this process simultaneously alters redox homeostasis and facilitates membrane-level lipid peroxidation. First, inflammatory stress can weaken antioxidant capacity: mastitis-related ferroptosis models link such stress to GSH/GPX4-axis impairment and lipid peroxidation (44). In parallel, neutrophil infiltration and respiratory bursts can generate sustained local oxidative pressure and neutrophil extracellular trap (NET)-associated inflammatory activity (45), increasing the demand on antioxidant systems while supporting antibacterial defense. Second, in several inflammatory models, pro-inflammatory cytokines influence System x_c^−^-related transport and antioxidant gene regulation, leading to restricted SLC7A11 expression or function, insufficient cystine supply, and impaired GSH synthesis. Under these conditions, even moderate oxidative load may lead to phospholipid peroxide accumulation because clearance capacity is inadequate (29, 44). Third, inflammatory signaling may reshape the expression of iron-metabolic genes, altering the balance among iron uptake, sequestration, and export. The net effect varies by cell type and temporal window, but in some contexts increased iron input or impaired iron export may push the reactive iron pool toward a more pro-peroxidative state (29, 44, 46). Thus, the early phase of mastitis should be evaluated not only by the initiation of inflammation, but also by whether inflammatory networks increase peroxidation pressure while eroding antioxidant reserve on the same time scale, thereby making membrane phospholipid peroxidation more likely to cross an irreversible threshold (44, 46).

### 3.2 Iron sources and iron availability: local iron-flux remodeling and expansion of the reactive iron pool

One prerequisite for ferroptosis is expansion of the labile iron pool (LIP) within a critical temporal window, providing catalytic conditions for chain lipid peroxidation. Changes in iron sources and iron availability within mastitic lesions may arise from tissue injury, heme-related metabolism, and immune-cell-mediated iron redistribution. In severely injured lesions with microhemorrhage, erythrocyte and hemoglobin release may become local iron sources. Heme handling and degradation pathways, including heme oxygenase-1 (HO-1), may not only release iron but also amplify oxidative pressure, increasing iron supply and peroxidation pressure simultaneously (47). In parallel, immune-cell infiltration remodels iron flux within inflammatory foci. Macrophages and other myeloid cells restrict pathogen access to iron, but they also alter iron uptake, sequestration, and release, leading to redistribution of iron among cell populations (17, 48). Importantly, host iron-withholding responses do not necessarily reduce iron risk in parenchymal cells. Under specific conditions, they may instead promote relative iron accumulation or LIP expansion in these cells, thereby increasing ferroptosis susceptibility (49). Consequently, iron-related changes in mammary tissue are often spatially heterogeneous: areas with dense immune infiltration, marked tissue injury, or active iron mobilization may lie closer to the ferroptotic threshold. Establishing tissue-level causal evidence will therefore require time-course and spatially resolved approaches rather than relying solely on averaged readouts from bulk tissue homogenates.

### 3.3 Metabolic reprogramming: restriction of the cysteine-GSH axis and structural shifts in the membrane-lipid substrate pool

Metabolic reprogramming in mastitis is important because it can simultaneously weaken clearance capacity and increase substrate susceptibility (50). On the clearance side, inflammatory and pathogen-related stress can affect cystine transport and SLC7A11-mediated cysteine/GSH supply, making the System x_c^−^–GSH–GPX4 axis described above more vulnerable to insufficiency under sustained oxidative load (28, 51). In mastitis-relevant host–pathogen models, pathogen stimulation has been associated with SLC7A11 suppression, GSH/GPX4-axis impairment, and increased lipid peroxidation, consistent with a possible sequence in which impaired peroxide clearance favors progressive lipid peroxide accumulation (51).

On the substrate side, structural shifts in lipid metabolism during inflammation can alter the proportion and distribution of PUFA-PLs within membrane phospholipids. Studies in lipid metabolism and ferroptosis show that PUFA supply, loading, and remodeling determine the quantity and type of substrates available for peroxidation; ACSL4- and LPCAT3-related processes are key to forming peroxidation-prone polyunsaturated fatty acid-containing phospholipids (PUFA-PLs) (10, 52). Recent work further suggests that phospholipid subclasses containing dual PUFA tails markedly increase ferroptosis sensitivity, indicating that the membrane lipidome itself can shift the threshold (18, 53). When clearance capacity declines while the substrate pool shifts toward greater peroxidation susceptibility, catalytic conditions supplied by iron homeostasis become more easily coupled in time with membrane-substrate vulnerability. The system is then more likely to remain in a state in which generation exceeds clearance and ultimately crosses the threshold (10, 50, 53).

### 3.4 Acute and chronic disease courses: differences in triggers, temporal windows, and pathological contribution

The triggers and pathological contribution of ferroptosis in mastitis are likely to vary with disease stage. At present, however, these differences are better regarded as a working model requiring further validation, rather than as conclusions systematically established in natural bovine cases. Current evidence suggests that acute mastitis is usually accompanied by stronger innate immune activation and neutrophil infiltration, with local oxidative load increasing rapidly over a short period (54–56). On this basis, ferroptosis-related injury, if present, would be expected to appear as an early and relatively narrow window of lipid-peroxidation pressure, rather than as a kinetic pattern already validated in natural bovine cases. Even before clearance systems are fully exhausted, phospholipid peroxides may accumulate rapidly and push membrane systems beyond an irreversible threshold. Acute inflammation is also characterized by the parallel activation of apoptosis, pyroptosis, necroptosis, and other regulated cell-death pathways. Thus, if a relatively prominent ferroptosis-related window exists, it is likely to appear earlier, be narrower in duration, and require careful sampling and pathway discrimination (57, 58).

By contrast, chronic or subclinical mastitis more often involves persistent low-grade inflammation, repeated microinjury, and failed repair, with immune-metabolic disturbance appearing as a long-term shift rather than a transient peak (59, 60). At this stage, ferroptosis-related changes may be better understood as a gradual lowering of the threshold. Cytokine profiles, somatic cell features, and milk ion and mineral indicators can remain persistently displaced from homeostasis in chronic lesions, suggesting that barrier fragility and functional burden accumulate slowly under low-grade inflammation (59, 60). In this setting, ferroptosis is less likely to appear as a single burst of extensive cell death and more likely to function as a sustained damage amplifier, continuously translating limited epithelial lipid peroxidation and structural injury into barrier disruption and inflammatory spread. Intercellular propagation mechanisms, if present, could further expand local lesions (61).

Accordingly, ferroptosis-related injury in acute versus chronic or subclinical mastitis should be viewed as stage-specific windows with different onset timing, duration, and evidentiary requirements, rather than as a single fixed and universally generalizable kinetic process (Figure 2). This distinction provides a temporal basis for analyzing how ferroptosis-related events may be translated into inflammatory amplification, barrier disruption, and lactation dysfunction. Validating such stage-specific kinetic models in naturally occurring bovine mastitis faces several practical obstacles. Natural cases are heterogeneous in pathogen identity, infection duration, disease severity, lactation stage, and prior treatment history, making it difficult to assign samples to defined disease windows. In addition, repeated sampling of the same mammary region across disease progression is rarely feasible in field settings, which limits the longitudinal and spatial resolution needed to test whether iron dysregulation, lipid peroxidation, defense-axis failure, and tissue injury occur in a defined temporal order.

## 4 Potential links between ferroptosis-related injury, inflammatory amplification, and functional dysfunction: oxidized lipid signals, death networks, and barrier endpoints

The previous sections outlined the core ferroptosis modules and how the mastitic microenvironment may lower the ferroptotic threshold. This section moves from upstream susceptibility mechanisms to downstream tissue consequences, asking how intracellular ferroptosis-related changes could be translated into tissue-level inflammatory amplification, barrier disruption, and functional impairment. If ferroptosis contributes mechanistically to mastitis, one of its key implications is that uncontrolled membrane phospholipid peroxidation may convert intracellular metabolic stress into a damage-amplifying process. This process may begin with membrane instability and be accompanied by the release of oxidized lipids and damage-associated molecular patterns (DAMPs), which can recruit and activate immune cells and affect blood–milk barrier integrity and mammary functional homeostasis. Current evidence supporting this chain, however, is not uniformly strong. Some evidence comes from pathogen-related mastitis models or mammary epithelial systems, whereas other evidence derives from chemical-stress models or broader studies of ferroptosis and inflammation. The aim here is therefore to distinguish directly supported relationships from biologically plausible mechanistic inferences and to consider their pathological relevance across temporal windows (62, 63).

### 4.1 Lipid peroxidation products and damage-associated molecular patterns: candidate mediators of inflammatory positive feedback

The most characteristic biochemical output of ferroptosis is peroxidation of polyunsaturated fatty acid-containing phospholipids and the generation of oxidized lipid products. These molecules are not only injury markers, but may also connect ferroptosis with innate immune activation. Recent reviews highlight oxidized phospholipids as bridges among ferroptosis, immunity, and inflammation, and ferroptosis itself is bidirectionally linked to NF-κB, MAPK, inflammasomes, and related pathways (64, 65). Research on immunogenic cell death further indicates that ferroptotic cells can release high-mobility group box 1 (HMGB1), adenosine triphosphate (ATP), and other DAMPs, shifting local immune responses from pathogen clearance toward damage amplification (66). These studies provide an important mechanistic background for understanding sustained inflammation in mastitis, but they do not themselves constitute direct causal evidence in bovine mastitis.

In the context of mastitis, current support comes from two main levels. The first is direct observation in pathogen-related models. Studies show that stimulation by *Klebsiella pneumoniae* or *Escherichia coli* (*E. coli*) in bovine mammary epithelial systems can be accompanied by iron accumulation, increased lipid peroxidation, and GPX4 suppression (8, 67). These findings indicate that, under pathogen-related stress, ferroptosis-like changes and mammary epithelial injury can occur together, providing evidence closer to the disease context for the possibility that oxidized lipid output contributes to local inflammatory amplification. The second level is mechanistic supporting evidence from mammary epithelial stress models. One commonly cited example comes from a non-pathogenic mammary epithelial stress model; because this model is chemically induced rather than pathogen-driven, it should be treated as epithelial-level mechanistic support rather than direct causal evidence in infectious mastitis. In this epithelial stress model, Ferrostatin-1 (Fer-1) reduced inflammatory cytokine release and improved the expression of barrier-related proteins, including zonula occludens-1 (ZO-1), occludin, and claudin-3 (62). Thus, this result supports a possible link between membrane lipid peroxidation and barrier dysfunction, but its comparability with pathogen-induced mastitis still requires direct experimental validation.

Taken together, current evidence supports oxidized lipids and DAMPs as candidate bridges linking ferroptosis to sustained inflammation and barrier injury in mastitis, rather than as components of a fixed causal pathway validated across models. Their pathological significance is likely to vary by pathogen background, disease stage, and cellular compartment, and should be tested in disease-relevant models.

### 4.2 Interactions with pyroptosis and necroptosis: cooperation, sequence, and dominant contexts

In highly inflammatory diseases such as mastitis, tissue injury is rarely attributable to a single mode of cell death. Instead, it is usually the result of multiple regulated cell-death programs acting together (68). Ferroptosis, pyroptosis, and necroptosis can be activated in the same pathological setting, and their relationship is more likely to involve dynamic cooperation or sequential amplification than mutual exclusion (57, 65). Consistent with the stage-specific framework outlined in Section 3.4, the relative balance among pyroptosis, necroptosis, and ferroptosis-related injury is likely to shift with pathogen background and disease phase, rather than following a fixed acute-to-chronic sequence (57, 65, 68–70).

Mechanistically, lipid peroxidation and inflammasomes can amplify each other bidirectionally. Restricting lipid peroxidation can reduce NOD-like receptor family pyrin domain-containing 3 (NLRP3)-related signaling and interleukin-1β (IL-1β) maturation, whereas inhibition of inflammasomes or their upstream integrative nodes may also alleviate oxidative load, iron imbalance, and propagation of membrane injury (65, 71, 72). Such crosstalk suggests that cell death in mastitis is more likely to follow a parallel and sequential network structure than a linear pathway dominated by one death program. The more relevant question is which pathway functions as the dominant damage-amplifying component under a given pathogen background and disease stage.

### 4.3 Epithelial barrier disruption and lactation failure: from molecular injury to production phenotypes

The potential significance of ferroptosis in bovine mastitis lies not only in its ability to induce mammary epithelial cell death, but also in its capacity to intensify epithelial barrier disruption and secretory dysfunction through lipid peroxidation, ultimately translating into reduced milk yield and altered milk quality (7, 73, 74). As discussed in Section 3.4, the tissue-level consequences of ferroptosis-related injury are likely to vary across disease stages and should therefore be interpreted within defined temporal windows. This section should be understood as describing a candidate translational chain from molecular injury to production phenotype, rather than a single pathway already proven by *in vivo* causal experiments.

In addition to barrier injury, lactation impairment is an important consequence of persistent mastitic damage. Milk synthesis depends on energy supply, redox homeostasis, lipid metabolism, and protein processing, and is also shaped by key epigenetic and transcriptional regulatory networks (75). In LPS-stimulated bovine mammary epithelial cell models, oxidative stress induction and disruption of lipid metabolism have been reported, thereby suppressing processes related to milk-fat synthesis (74). In addition, mastitis-related studies suggest that endoplasmic reticulum stress can be accompanied by lipid-metabolic disturbance and cellular injury, further weakening the secretory homeostasis of mammary epithelial cells (76). Ferroptosis-related events may therefore contribute to mammary epithelial dysfunction through a candidate chain linking membrane injury, metabolic imbalance, and secretory impairment, although its *in vivo* causal status still requires further validation in infection models and naturally occurring cases (7, 74, 76).

**Figure 3:**
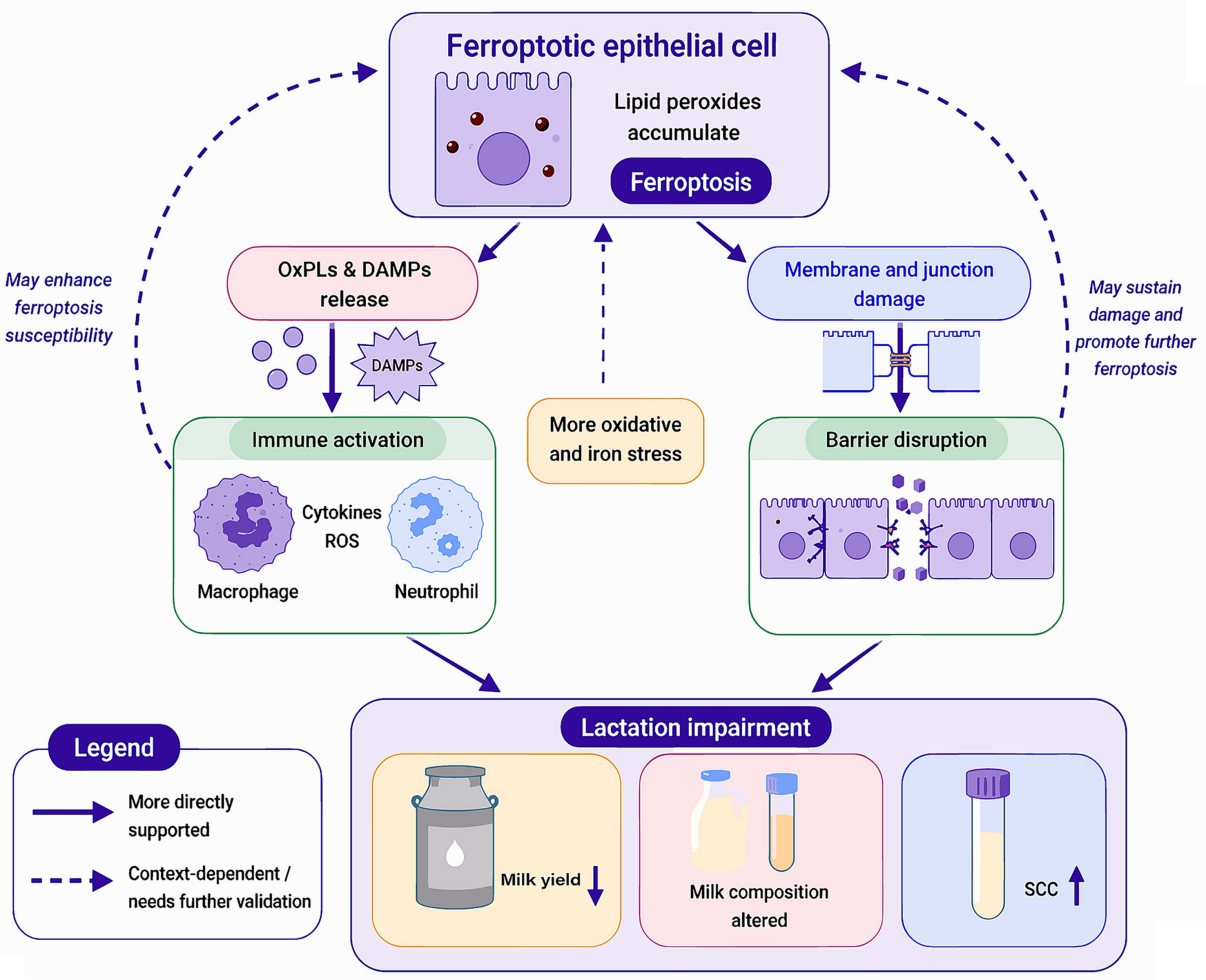
Integrated pathway linking ferroptosis to inflammatory amplification and mammary dysfunction. This figure illustrates how ferroptosis-related membrane phospholipid peroxidation may be translated into inflammatory amplification, barrier disruption, and impaired lactation. The central panel depicts mammary epithelial cells undergoing ferroptosis-related membrane lipid peroxidation. The left branch indicates how oxidized lipids and DAMPs may activate immune cells and increase local oxidative and inflammatory pressure, whereas the right branch indicates how membrane and tight-junction injury can increase blood-milk barrier permeability. The lower panel shows outcomes such as reduced milk yield, altered milk composition, and increased SCC. Solid arrows indicate relatively more direct support, whereas dashed arrows indicate context-dependent links that require further validation.

These molecular and cellular changes are ultimately reflected in production phenotypes. Elevated SCC is one of the most established milk-based indicators of mastitis-associated inflammation and epithelial injury. At the herd level, mastitis has cumulative and persistent adverse effects on milk yield and lactose content, indicating that epithelial barrier damage and impaired synthetic function are not merely transient changes but may cause sustained production losses (2). Accordingly, evaluation of ferroptosis-directed interventions should not be limited to molecular or histological readouts, but should also include SCC, milk electrical conductivity, milk composition, and milk yield (2, 77). Within the framework of this review, ferroptosis is most relevant as a potential link between uncontrolled membrane phospholipid peroxidation and a continuum of inflammatory amplification, barrier disruption, and impaired lactation. This candidate integrative pathway is summarized in Figure 3.

## 5 Building the evidence chain: multilevel strategies for rigorously identifying ferroptosis in mastitis

The evidence framework outlined above must be translated into study designs that are executable, comparable, and causally informative in the complex disease setting of mastitis. Whereas Box 1 defines standards of claim strength, this section focuses on implementation: stepwise model selection, temporal-window design, tissue and cellular compartment resolution, marker-panel configuration, intervention experiments, and confounder control. Together, these elements can move correlation-based observations toward more reliable mechanistic inference.

### 5.1 Model hierarchy: a progressive path from mechanistic analysis to pathological validation

Model selection is crucial for validating ferroptosis mechanisms, and a stepwise progression from simple to complex systems is most appropriate. Questions related to acute disease are best addressed first in mammary epithelial cell models and short-term stimulation systems, in which LPS, pathogen stimulation, iron loading, and cysteine availability can be manipulated to determine whether early membrane lipid peroxidation, defense-axis impairment, and injury rescue occur in parallel. Chronic or subclinical questions require longer-term stimulation models, tissue models, and natural cases to evaluate threshold lowering, failed repair, and functional endpoints under persistent low-grade inflammation. Studies have shown that Ferrostatin-1 reduces Fe^2+^ accumulation, oxidative stress, and inflammatory injury in LPS-treated goat mammary epithelial cells, and that Ferrostatin-1 or iron-chelation intervention can attenuate lipid peroxidation and tissue injury in *Staphylococcus aureus*-induced mastitis models, suggesting that iron-dependent injury may participate in mastitis pathogenesis (78, 79). However, *in vitro* models can only test mechanistic feasibility; they cannot recapitulate the tissue microenvironment, immune-cell infiltration, or systemic iron-metabolic changes of mastitis, and therefore cannot replace *in vivo* pathological evidence (78, 79).

To improve translational relevance, evidence should be extended to *in vivo* models and naturally occurring bovine cases. Experimental infection models allow relationships between ferroptosis and mastitis progression to be examined under relatively controlled conditions. Naturally occurring cases are better suited for determining whether ferroptosis-related molecular markers are stably associated with clinical outcomes such as SCC, changes in milk composition, and milk-yield loss. Because mastitis can persistently reduce milk yield and lactose content, these production traits are appropriate endpoints for evaluating the clinical relevance of ferroptosis (2). Natural case samples, however, are highly heterogeneous. Molecular readouts from bulk tissue homogenates often integrate contributions from epithelial cells, infiltrating immune cells, and stromal components. Without cell-type resolution, ferroptosis markers may be diluted or misinterpreted (80). In such samples, laser-capture microdissection and other methods that resolve cellular or tissue compartments should be incorporated where possible to restrict readouts to mammary epithelium or defined immune-cell populations. Such spatial and cell-type-resolved strategies can improve data accuracy and interpretability and provide a more reliable basis for subsequent clinical studies. The predominance of homogenized tissue analyses in the current literature likely reflects practical constraints rather than methodological preference. Laser-capture microdissection, fluorescence-activated cell sorting, spatial transcriptomics, and compartment-resolved imaging require fresh or optimally preserved tissue, specialized equipment, and longer processing times than bulk homogenization, conditions that are difficult to meet in field-collected mastitis samples. As a result, ferroptosis-related markers measured from whole-tissue lysates may reflect changes in cellular composition, immune-cell infiltration, epithelial injury, or stromal remodeling, rather than ferroptotic activity within a defined cell type. Establishing cell-type-resolved evidence is therefore not merely a technical refinement but a prerequisite for defining the biological meaning and intervention target of ferroptosis in mastitis (80).

### 5.2 Marker layer: convergent multidimensional evidence and specificity of interpretation

Reliable identification of ferroptosis cannot rely on a single indicator. Evidence should integrate iron-metabolic abnormalities, phospholipid-specific peroxidation, and impairment of core defense systems (81, 82). Acute studies require higher temporal resolution, ideally demonstrating that LIP expansion, membrane lipid peroxidation, and GPX4/GSH-axis impairment occur before tissue disintegration. Chronic or subclinical studies should assess whether these indicators remain displaced from homeostasis over time and are associated with endpoints such as barrier fragility, reduced milk yield, or altered milk composition. First, iron dependence of the injury process should be demonstrated. This is most directly reflected by LIP expansion or dynamic change, which can be detected using specific fluorescent probes such as FerroOrange (17, 82). Changes in ferritin or transferrin receptor expression may serve as supportive evidence, but because they are influenced by multiple pathways in inflammatory settings, they lack sufficient specificity to serve as key evidence alone (17, 81). More compelling evidence comes from functional validation: if an iron chelator such as deferoxamine markedly reduces cellular injury, the involvement of catalytic iron in the pathological chain is more directly supported (17, 81).

### 5.3 Causal layer: intervention experiments and dynamic analysis to establish mechanistic necessity

Correlative observations can support a hypothesis, but causal inference requires intervention. To demonstrate a mechanistic contribution of ferroptosis in mastitis, intervention experiments must establish a causal chain. In acute disease, the key question is whether early restriction of iron-dependent membrane lipid peroxidation can preserve barrier integrity and reduce structural damage before the inflammatory peak is established. In chronic or subclinical disease, the focus shifts to whether longer-term intervention can reduce persistent oxidative injury, improve repair, and produce better production endpoints. At the pharmacological-rescue level, reliance on a single inhibitor is insufficient. A more robust strategy is to combine interventions with different mechanisms, such as lipid-radical scavengers (for example, Ferrostatin-1 or Liproxstatin-1) and iron chelators (for example, deferoxamine). If both types of intervention consistently reduce cell death or tissue injury, whereas inhibitors of apoptosis, pyroptosis, or other death pathways are ineffective, the causal chain becomes more convincing (83, 84).

Higher-grade evidence comes from genetic intervention. In cellular models, GPX4 overexpression or ACSL4 knockdown should, in principle, alter sensitivity to ferroptosis-inducing conditions. At the animal level, conditional genetic approaches, such as mammary epithelial cell-specific GPX4 knockout models, would provide stronger evidence for the *in vivo* necessity of ferroptosis if they alter susceptibility to mastitis or the type of pathology observed (85, 86). Time-course analysis is also indispensable for causal inference. Ferroptosis is a dynamic threshold event, and dense sampling across multiple time points can help resolve the order of key events. Ideally, iron-metabolic disturbance should precede marked lipid peroxidation, which should in turn precede large-scale cell disintegration and the inflammatory peak. Only when membrane lipid peroxidation is shown to occur before irreversible structural injury can it be more strongly interpreted as a driver rather than a secondary phenomenon (87).

### 5.4 Misclassification and confounding: key controls in a complex pathological background

Identifying ferroptosis in mastitis also requires careful handling of key confounders. This is important in both acute and chronic stages, although the emphasis differs. In acute disease, delayed sampling and mixed injury caused by parallel activation of multiple death pathways in a strongly inflammatory background are major risks. In chronic or subclinical disease, long-term drug exposure, differences in metabolic state, and tissue heterogeneity may confound low-grade sustained signals. Some clinical treatments, especially antibiotics and interventions with anti-inflammatory or antioxidant activity, may directly or indirectly affect redox balance, iron metabolism, and cell-death pathways, thereby interfering with ferroptosis attribution (88, 89). Study design and data analysis should therefore record drug use systematically and evaluate its potential effects where possible. Markers of pyroptosis and necroptosis should also be measured in parallel to more accurately determine the relative contribution of each pathway (70, 90, 91). Sampling time is equally critical. Sampling too early may capture only defensive stress signals, whereas sampling too late may be confounded by secondary necrosis. Dynamic analysis across multiple time points remains essential for reducing misclassification, especially when ferroptosis signals are measured in a mixed inflammatory background (87).

The current evidence is unevenly distributed across pathogen models, disease stages, and cellular compartments. Table 1 summarizes where ferroptosis is supported by pathogen-related evidence, mechanistic inference, or indirect disease-context data.

**Table 1:** Context-dependent evidence map for ferroptosis-related injury in bovine mastitis.

Context	Main ferroptosis-related evidence	Main compartment	Evidence status	Key references
Natural or clinical bovine mastitis	HMOX1/FTH1-related iron dysregulation, lipid peroxidation, GPX4-axis restriction, and association with mammary injury	Mammary tissue; epithelial contribution inferred	Disease-associated evidence; causal and cell-type-resolved data remain limited	(9)
*Klebsiella pneumoniae* mastitis models	Iron accumulation, Nrf2/xCT/GPX4 inhibition, NCOA4-mediated ferritinophagy, lipid peroxidation, and lactation dysfunction	Mammary epithelial cells and tissue	Relatively strong pathogen-related mechanistic evidence; finer time-course and clearance endpoints are needed	(8, 20, 63)
*Escherichia coli* stimulation or infection	Iron accumulation, GPX4 suppression, lipid peroxidation, mitophagy-related signaling, and epithelial ferroptosis-like injury	Bovine mammary epithelial cells	Direct pathogen-related epithelial evidence; *in vivo* causal role remains insufficiently defined	(67)
*Staphylococcus aureus* models	SLC7A11 suppression, GSH/GPX4 impairment, ferroptosis-related gene signatures, and SIRT1/p53/SLC7A11 signaling	Mammary epithelial cells; some tissue-level evidence	Pathogen-related mechanistic evidence; stage-specific contribution remains unresolved	(29, 51, 79)
LPS or inflammatory stimulation	Fe^2+^ accumulation, oxidative injury, GSH/GPX4 impairment, lipid peroxidation, and partial rescue by Fer-1-related interventions	Mammary epithelial cell models	Mechanistic support; does not fully reproduce infection complexity or pathogen clearance	(44, 78)
Non-pathogen epithelial stress	Lipid peroxidation, inflammatory cytokine release, tight-junction disruption, and Fer-1 rescue	Bovine mammary epithelial cells	Epithelial-level mechanistic support; not direct infectious-mastitis evidence	(62)
Regulated cell-death crosstalk	Interactions among ferroptosis, pyroptosis, necroptosis, NLRP3 signaling, IL-1β maturation, and membrane injury	Tissue-level and network inference	Indirect mechanistic support; dominant pathway must be tested by pathogen and stage	(57, 58, 65, 68–72)
Chronic or subclinical mastitis	Persistent cytokine displacement, altered somatic cell and milk mineral traits; sustained lipid peroxidation remains hypothetical	Mammary tissue; epithelial and immune compartments unresolved	Indirect disease-context support; direct ferroptosis time-course evidence remains limited	(59–61)
Milk-based molecular remodeling	Milk metabolomic, proteomic, and multi-omic remodeling; potential integration with oxidized lipids, GPX4 activity, SCC, and milk-yield trajectory	Milk matrix; mixed epithelial, immune, and secretory outputs	Emerging translational evidence; not yet a ferroptosis-specific panel	(96–99)

## 6 Controversies and boundaries: ferroptosis contribution, target cells, and immune trade-offs

Although ferroptosis provides an interpretable and testable framework for tissue injury in mastitis, its relative contribution across pathogen spectra, disease stages, and cellular compartments remains uncertain. Current discussion has moved from whether ferroptosis is associated with mastitis to whether it contributes to damage amplification, but a stable spatiotemporal causal framework has not yet been established. The critical question is no longer simply whether ferroptosis “exists” in mastitis, but three more specific questions: how large is its contribution under different pathogens and temporal windows; which target cells drive injury—mammary epithelial cells, immune cells, or sequential contributions from both; and can ferroptosis-related damage be limited without compromising pathogen clearance while improving tissue repair and production outcomes? The following subsections discuss these major controversies and evidence boundaries.

### 6.1 Causality: driver, amplifier, or bystander?

The position of ferroptosis in the pathological chain of mastitis is inherently context-dependent and cannot be generalized. Studies have reported iron accumulation, enhanced lipid peroxidation, and restriction of GPX4/SLC7A11 defenses in mammary tissue from clinical mastitis cows and in models involving *Staphylococcus aureus*, *E. coli*, or *Klebsiella pneumoniae*. More importantly, in some models, interfering with these processes alleviates mammary epithelial injury, inflammatory amplification, and lactation dysfunction, suggesting that ferroptosis is not merely a terminal phenomenon in certain settings, but may contribute to tissue-injury amplification during defined pathogen-related windows (20, 79). On the other hand, mastitis remains an inflammatory cascade driven by pathogen-associated molecular patterns. At the inflammatory peak, ROS input, iron dyshomeostasis, and multiple regulated cell-death pathways often coexist. Thus, ferroptosis signals detected at the tissue level may also represent one cell-death program selected under a highly oxidative microenvironment, rather than the sole upstream driver.

A more defensible interpretation is that ferroptosis acts differently across pathological contexts. Under some pathogen backgrounds and temporal windows, it may serve as a damage-amplifying node; in others, it may mainly reflect intense inflammation and oxidative injury. Progress will require more than additional cross-sectional correlations. Pathogen stratification, high-resolution temporal sampling, spatial localization, and causal intervention should be integrated to determine when ferroptosis occurs, which cell type it affects, and whether it is required for tissue injury (51, 92). Only then can its mechanistic position in mastitis be defined with greater confidence.

### 6.2 Cell-type specificity: dual roles of epithelial and immune cells

The most direct evidence in bovine mastitis currently centers on mammary epithelial cells. Overall, the available findings support a relatively cautious conclusion: epithelial ferroptosis is more directly linked to blood-milk barrier disruption, inflammatory amplification, and impaired lactation, which explains why many intervention studies focus on epithelial iron homeostasis, membrane lipid peroxidation, and the GPX4 axis (51, 67, 93). Based on current evidence, mammary epithelial cells remain the most well-supported core target cell type.

Mastitic lesions, however, are not purely epithelial. Macrophages, neutrophils, and other infiltrating immune cells also reside in a microenvironment characterized by high oxidative load, iron redistribution, and active inflammatory signaling. Mechanistically, these cells may undergo ferroptosis or be substantially affected by ferroptosis-related signals. For bovine mastitis specifically, however, this area remains largely inferential and is informed mainly by broader infection-immunology studies. Available evidence suggests that, in contexts of high oxidative load and iron redistribution, ferroptosis in immune cells may alter cytokine profiles, phagocytic clearance, and handling of necrotic debris, and may bidirectionally influence host defense and tissue injury (93, 94). Thus, the pathological significance of ferroptosis lies not in broadly demonstrating that it occurs, but in identifying which cells are affected, where they are located, and when these events occur.

From a study-design perspective, the critical advance will not come from adding more averaged whole-tissue readouts, but from using spatial localization, cell sorting, and cell-type-specific interventions to identify the cell population that drives injury. Only when the relative contributions of epithelial cells and immune cells across temporal windows are resolved can the biological meaning and intervention value of ferroptosis in mastitis be defined more precisely (51, 93).

### 6.3 Immune trade-offs: could suppressing ferroptosis impair pathogen clearance?

Ferroptosis-directed strategies are host-directed anti-injury interventions rather than direct antibacterial treatments, and their value should not be equated with improved pathogen clearance. Excessive or premature suppression could impair clearance by altering iron homeostasis, immune-cell function, or inflammatory timing. The key question is therefore context-specific: under which pathogen background, temporal window, and cellular compartment do the benefits of ferroptosis inhibition outweigh its risks (93, 94)? A benefit–risk framework for ferroptosis-directed intervention should therefore consider at least three competing dimensions. First, ferroptosis-related pathways in neutrophils and macrophages may participate in antimicrobial responses and pathogen control; suppressing these pathways during active infection could reduce bactericidal efficiency and prolong pathogen persistence (95). Second, uncontrolled epithelial ferroptosis may amplify barrier disruption, inflammatory signaling, and lactation failure even after pathogen burden has declined. Third, the relative weight of these effects is expected to shift with disease stage: during the acute inflammatory peak, preserving pathogen clearance may outweigh tissue-protection gains, whereas in chronic or subclinical disease, where persistent epithelial injury and failed repair may dominate, the balance may shift toward epithelial protection. Quantifying this trade-off will require studies that report pathogen load, clearance kinetics, histological injury, and production endpoints in parallel. Therefore, the net benefit of ferroptosis-directed intervention should be tested within a dual-endpoint framework that includes both pathogen-related and tissue-related outcomes (67, 79, 96, 97).

The translational relevance of ferroptosis inhibition therefore depends on context. Future studies should adopt pathogen-, time-window-, and cell-type-stratified designs, report pathogen outcomes, barrier and histological endpoints, and production endpoints in parallel, and distinguish epithelial-protective benefits from immune-defense costs. Ferroptosis can be considered a translatable target only if intervention improves tissue injury and functional recovery without substantially impairing pathogen clearance (79, 92–94, 96, 97).

## 7 From mechanism to translation: stratified assessment and intervention boundaries for targeting ferroptosis

Ferroptosis offers a mechanistic entry point for controlling tissue injury in mastitis, but translation requires more than reducing a molecular marker. Two questions are central: can the pathogen background, temporal window, and host injury state suitable for intervention be identified; and can a given strategy produce interpretable tissue-level and functional benefits? This section first considers the potential value of milk biomarker panels for stratified assessment, and then discusses three intervention paths in order of evidence strength and translational maturity: tool-based interventions for defining mechanistic contribution, host-support strategies compatible with herd-level management, and exploratory approaches involving local delivery systems and natural products.

### 7.1 Milk biomarkers and stratified assessment: a bridge from mechanistic research to clinical management

Among translational questions, one of the more realistic directions is to develop reproducible and interpretable milk-based biomarker panels related to ferroptosis. Rather than pursuing a single “specific molecule,” a more practical strategy is to integrate multiple readouts reflecting iron availability or iron flux, membrane lipid peroxidation, and restricted antioxidant defense, and to interpret them together with SCC, milk electrical conductivity, milk composition, and the trajectory of milk-yield recovery. Recent milk metabolomic, proteomic, and multi-omic studies show that both clinical and subclinical mastitis are associated with stable and detectable molecular remodeling, and that milk-response profiles differ by pathogen. These findings provide a basis for developing candidate frameworks for stratified assessment of ferroptosis-related injury, but they are still insufficient to define a ferroptosis-specific panel (96–99).

Several barriers must be addressed before such panels can be used clinically. Milk is a highly complex biological matrix. Milking interval, storage conditions, milk-fat composition, and inflammatory exudation can all substantially affect lipid and protein oxidation readouts. Thus, even markers that are mechanistically related to ferroptosis may not translate directly into stable and comparable clinical readouts. A more feasible near-term goal is not to claim that a milk-based ferroptosis stratification tool is already mature, but to integrate milk oxidized-lipid profiles, GPX4/antioxidant activity readouts, SCC, and milk-yield trajectories in natural case cohorts and to test whether this multi-dimensional panel predicts recovery speed, recurrence risk, or therapeutic benefit after standardized pre-analytical handling and repeated validation (96–99).

This direction is therefore best understood as a bridge from mechanistic research to clinical management. Building such a framework—one that connects pathogen background, temporal window, and functional outcome—matters more than identifying any single ferroptosis-specific marker. If validated in prospective cohorts, such a framework could allow ferroptosis research to move beyond tissue and molecular mechanisms toward more actionable mastitis-management scenarios.

### 7.2 Mechanism-validating interventions: the tool-like role of defining ferroptosis contribution

At the current stage, lipid peroxidation chain-reaction inhibitors such as Ferrostatin-1 (Fer-1) and Liproxstatin-1 (Lip-1), as well as iron-restriction strategies such as deferoxamine (DFO), are better regarded as mechanistic probes than as mature interventions ready for on-farm use. Their principal value is to test a causal chain: whether limiting iron-dependent membrane phospholipid peroxidation can reduce structural injury. This, in turn, can help determine the relative contribution of ferroptosis in specific models, pathogens, and disease stages (100, 101).

From a stage-stratified perspective, the acute phase requires attention to whether early restriction of membrane lipid peroxidation can preserve barrier integrity and reduce structural damage without substantially impairing pathogen clearance. Chronic or subclinical stages are better suited for examining whether sustained limitation of oxidative injury facilitates repair and improves functional recovery. More informative than a simple reduction in a single molecular marker is whether intervention produces interpretable effects across endpoint layers. If Fer-1, Lip-1, or DFO consistently reduces membrane lipid peroxidation and improves tight junctions, histological injury, or barrier-related endpoints, while having only limited direct effects on inflammatory intensity, this would support a role for ferroptosis in structural damage amplification. Conversely, if inflammatory markers decline but lipid peroxidation and barrier outcomes improve little, ferroptosis may not be the dominant execution pathway, or the intervention window, exposure level, or target engagement may be inadequate (62, 102).

Accordingly, these agents are best treated as tool interventions for clarifying pathological attribution, prioritizing targets, and defining effective temporal windows, rather than as clinically mature therapies. Only when pathogen-related outcomes, histological endpoints, and production endpoints show concordant benefit should such strategies be advanced into more realistic translational discussions.

### 7.3 Implementable nutrition and management strategies: reinforcing antioxidant networks and the membrane-lipid substrate pool

Nutritional and management approaches are generally more practical than new chemical entities for strengthening anti-peroxidation reserve and metabolic homeostasis in the mammary gland at the herd level. These approaches should not be framed as direct equivalents of ferroptosis-targeted therapy. Rather, they are best viewed as mechanism-informed host-support strategies that enhance clearance capacity, reduce substrate susceptibility, and alleviate inflammatory and metabolic triggers during high-risk windows (103).

In acute disease, nutritional strategies are more appropriate as adjunctive support alongside anti-infective treatment, with the aim of maintaining GSH supply, slowing membrane lipid peroxidation, and preserving anti-injury reserve in the epithelium. Chronic or subclinical stages may represent a more suitable application window. A combined approach centered on chain-termination support, maintenance of GSH supply, and micronutrient support may help slow persistent threshold lowering, promote repair, and reduce recurrence (103–106). With respect to substrate susceptibility, dietary fatty acid composition may affect peroxidation sensitivity by altering membrane phospholipid composition. Ferroptosis studies show that polyunsaturated fatty acid (PUFA) enrichment increases susceptibility to membrane lipid peroxidation, whereas monounsaturated fatty acids (MUFAs) exert relatively protective effects (10). In dairy cows, dietary regimens can also influence mammary oxidative status and milk-fat synthesis, supporting the broader relevance of nutritional modulation at the herd level (107). Fatty acid strategies are therefore better positioned for prevention during high-risk windows and reinforcement during recovery than as replacements for anti-infective treatment during acute infection.

Heat stress, periparturient metabolic pressure, and immune fluctuations can all increase triggering intensity and amplify oxidative stress. Stabilizing metabolic status, reducing environmental stress, and optimizing herd management are therefore prerequisites for these support strategies to be effective (108–110). When ferroptosis is viewed as a candidate node in host-damage amplification, systematic regulation of redox homeostasis, membrane-lipid substrate pools, and metabolic stress represents an implementable herd-level path for protecting mammary tissue, even before ferroptosis-specific evidence in mastitis is fully consolidated.

### 7.4 Local delivery systems and natural products: exploratory routes to improve local target engagement

In lactating animals, systemic administration is constrained by drug residues, safety, and cost. Intramammary administration therefore provides a more realistic delivery route for ferroptosis-related interventions. Its main advantage is increased exposure near the lesion and reduced systemic distribution, making it more likely to balance local efficacy with systemic risk (111, 112). At present, however, this direction should be viewed as an exploratory translational route rather than a mature strategy with well-defined application boundaries.

Stage should also guide local delivery. In acute disease, the ideal goal is to reduce oxidative injury in adjacent epithelium as early as possible on the basis of infection control, while minimizing interference with pathogen clearance. In chronic or subclinical disease, the emphasis shifts toward prolonging local exposure, promoting barrier repair, and reducing recurrence. Candidate selection should not focus only on dosage form; it should examine whether the evidence chain is complete. At appropriate doses, candidates should affect membrane lipid peroxidation outputs and key defense or substrate axes, such as GPX4/GSH, SLC7A11, ACSL4-related lipid remodeling, or iron availability, and should align with tissue-level barrier outcomes and farm-relevant endpoints (113).

Although natural products often have broad antioxidant and anti-inflammatory backgrounds, they are best categorized by mechanistic clusters: chain-terminating molecules that block the propagation of lipid peroxidation, molecules that enhance GSH supply or sulfur metabolism, molecules that buffer iron-induced oxidative reactions, and molecules that reduce inflammation-oxidation coupling (113). For these candidates, the key issue is not whether they have antioxidant activity in general, but whether, in the mastitis context, they generate a complete evidence chain linking ferroptosis-related readouts with histological improvement and production endpoints.

Local sustained-release or adhesive delivery systems, including liposomes, nanoparticles, and gels, may prolong effective exposure and reduce dosing frequency. However, local irritation, formulation stability, milk residues, and withdrawal periods require systematic assessment (112, 114–116). Rumen metabolism and biotransformation in ruminants, as well as the effects of dietary antioxidants and phenolic compounds on milk and dairy-product composition, should also be included in translational evaluation (117). This route may eventually support more production-compatible intervention formats, but its current evidence level remains below that of mechanism-validating interventions and host-support strategies.

Because the strategies discussed above differ in evidence strength and field applicability, Table 2 summarizes their translational maturity and the boundaries of the claims that can currently be made.

**Table 2:** Translational maturity and application boundaries of ferroptosis-directed strategies in mastitis.

Strategy	Main role	Best-fit use scenario	Current status and boundary	Key references
Milk biomarker panels	Stratified assessment and prognosis	Monitoring recovery, recurrence risk, and therapeutic benefit	Candidate stratification tool; not an established ferroptosis-specific diagnostic panel	(96–99)
Fer-1, Lip-1, and deferoxamine	Mechanistic validation of iron-dependent lipid peroxidation	Experimental acute or model-specific settings	Mechanistic probes; not mature on-farm therapies	(78, 79, 83, 84, 100, 101)
GPX4, ACSL4, NCOA4 manipulation	Causal attribution and pathway dissection	Controlled cellular or animal models	High mechanistic value; limited field applicability	(18–20, 85, 87)
Antioxidant and GSH-supportive nutrition	Reinforcement of redox reserve	High-risk periods, chronic/subclinical disease, and recovery	Mechanism-informed supportive management; pathway specificity remains limited	(16, 32, 103–110)
Fatty-acid or membrane-lipid modulation	Reduction of peroxidation susceptibility	Prevention and recovery reinforcement	Hypothesis-driven nutritional strategy; mastitis-specific ferroptosis evidence remains limited	(10, 31, 52, 53, 107)
Intramammary local delivery	Improved local target engagement	Model-dependent; potentially chronic/subclinical or recovery settings	Exploratory delivery route; residue, withdrawal period, irritation, and regulation must be addressed	(111–116)
Natural products or bioactive compounds	Modulation of lipid peroxidation, GSH supply, iron-driven oxidation, or inflammation–oxidation coupling	Adjunctive support or formulation development	Candidate compound classes; broad antioxidant activity is not equivalent to ferroptosis specificity	(113, 117)
Pathogen–mechanism–function endpoint design	Evaluation of net translational benefit	Intervention studies aiming at clinical relevance	Recommended validation framework before strong translational claims	(2, 77, 92–94, 96, 97)

## 8 Conclusion

Taken together, the available evidence supports a cautious conclusion: ferroptosis is best regarded as a candidate mechanistic framework for host-damage amplification in mastitis, rather than as a universal execution pathway established across pathogens, disease stages, and cellular compartments. Its contribution is likely to vary with pathogen background, disease stage, cellular compartment, and host condition. Iron dyshomeostasis, enhanced membrane lipid peroxidation, and restriction of the GSH/GPX4 defense system can, in specific contexts, lower the ferroptotic threshold of mammary epithelial cells and link this shift to barrier disruption, inflammatory amplification, and impaired lactation. Whether these changes are necessary for disease progression, which cellular compartments are primarily involved, and whether ferroptosis can be targeted safely without compromising pathogen clearance remain the central questions for future translation.

The next stage of research should move beyond broadly demonstrating that ferroptosis is detectable in mastitis and instead define its relative contribution, spatiotemporal localization, and application boundaries. Specifically, pathogen-stratified and densely sampled time-course models are needed to determine the temporal windows in which ferroptosis carries greater weight and to compare its relative contribution with parallel death pathways such as pyroptosis and necroptosis. Spatial localization, cell sorting, and cell-type-specific interventions should be used to clarify whether ferroptosis occurs mainly in mammary epithelial cells, infiltrating immune cells, or sequentially in both across disease stages. Pathogen-related, histological, and functional endpoints should be reported in parallel to test whether targeting ferroptosis can protect tissue without weakening pathogen clearance.

From a study-design perspective, the highest priority is to build time-course infection models stratified by dominant pathogens, covering acute through chronic or subclinical stages, while simultaneously resolving mammary epithelial and immune-cell compartments. Readouts should include the labile iron pool, membrane lipid peroxidation, SLC7A11/GPX4 defenses, parallel regulated cell-death pathways, and pathogen outcomes. Intervention studies should combine lipid-radical scavengers, iron chelators, and inhibitors of intersecting pathways, and should incorporate blood-milk barrier integrity, SCC, milk composition, and milk yield into endpoint systems. Only within this integrated pathogen-mechanism-function design can the causal coordinates and translational value of ferroptosis be defined more reliably.

The priority is therefore to move from detecting ferroptosis to defining the conditions under which it becomes pathologically indispensable. If this can be achieved, milk biomarker panels, mechanism-informed nutritional management, and local delivery interventions could progress from conceptual prospects to stratified management strategies with clearer application boundaries.
